# Raman Spectroscopy and Machine-Learning for Early Detection of Bacterial Canker of Tomato: The Asymptomatic Disease Condition

**DOI:** 10.3390/plants10081542

**Published:** 2021-07-28

**Authors:** Moisés Roberto Vallejo-Pérez, Jesús Antonio Sosa-Herrera, Hugo Ricardo Navarro-Contreras, Luz Gabriela Álvarez-Preciado, Ángel Gabriel Rodríguez-Vázquez, José Pablo Lara-Ávila

**Affiliations:** 1 Consejo Nacional de Ciencia y Tecnología-Universidad Autónoma de San Luis Potosí, CIACYT, Alvaro Obregon 64, Col. Centro, San Luis Potosí 78000, Mexico; 2 Coordinación para la Innovación y la Aplicación de la Ciencia y la Tecnología (CIACYT), Universidad Autónoma de San Luis Potosí, Av. Sierra Leona 550, Col Lomas 2a. Sección, San Luis Potosí 78210, Mexico; hnavarro@uaslp.mx (H.R.N.-C.); luzgaal@hotmail.com (L.G.Á.-P.); angel.rodriguez@uaslp.mx (Á.G.R.-V.); 3 Consejo Nacional de Ciencia y Tecnología-Centro de Investigación en Ciencias de Información Geoespacial A. C., Laboratorio Nacional de Geointeligencia, Aguascalientes 20313, Mexico; jasosahe@conacyt.mx; 4 Facultad de Agronomía y Veterinaria, Universidad Autónoma de San Luis Potosí, Km. 14.5 Carretera San Luis Potosí, Matehuala, Ejido Palma de la Cruz, Soledad de Graciano Sánchez, San Luis Potosí 78321, Mexico; pablo.lara@uaslp.mx

**Keywords:** *Clavibacter michiganensis* subsp. *michiganensis*, plant disease surveillance, precision farming, principal component analysis (PCA), multilayer perceptron (MLP), linear discriminant analysis (LDA)

## Abstract

Bacterial canker of tomato is caused by *Clavibacter michiganensis* subsp. *michiganensis* (Cmm). The disease is highly destructive, because it produces latent asymptomatic infections that favor contagion rates. The present research aims consisted on the implementation of Raman spectroscopy (RS) and machine-learning spectral analysis as a method for the early disease detection. Raman spectra were obtained from infected asymptomatic tomato plants (BCTo) and healthy controls (HTo) with 785 nm excitation laser micro-Raman spectrometer. Spectral data were normalized and processed by principal component analysis (PCA), then the classifiers algorithms multilayer perceptron (PCA + MLP) and linear discriminant analysis (PCA + LDA) were implemented. Bacterial isolation and identification (16S rRNA gene sequencing) were realized of each plant studied. The Raman spectra obtained from tomato leaf samples of HTo and BCTo exhibited peaks associated to cellular components, and the most prominent vibrational bands were assigned to carbohydrates, carotenoids, chlorophyll, and phenolic compounds. Biochemical changes were also detectable in the Raman spectral patterns. Raman bands associated with triterpenoids and flavonoids compounds can be considered as indicators of Cmm infection during the asymptomatic stage. RS is an efficient, fast and reliable technology to differentiate the tomato health condition (BCTo or HTo). The analytical method showed high performance values of sensitivity, specificity and accuracy, among others.

## 1. Introduction

The world population is rising continuously, therefore, it is essential to maintain an adequate food supply in order to satisfy the nutritional requirements of people living in urban and rural areas. The increase of crop productivity, without causing farmland degradation and overexploitation of natural resources poses a great challenge to producers. The adoption of improved agricultural practices contributes to increase crop efficiency, but food security is threatened by outbreaks of pests and plant diseases [1]. Therefore, highly efficient technologies applied for monitoring plant health are necessary, and those that provide an early detection of potential phytosanitary problems are strongly desired [2].

Tomato (*Solanum lycopersicum* L.) cultivars can be infected by *Clavibacter michiganensis* subsp. *michiganensis* (Cmm); a plant pathogenic actinomycete considered to be the causal agent of bacterial canker of tomato (BCTo). Cmm is directly responsible of important economic losses in tomato-growing areas worldwide and it is classified as a quarantine organism [3]. Potential yield losses can be severe (20–84%), but they vary according to year, phenological stage of host infection, cultivar, cultural practices, etc. [4]. There are no Cmm-resistant tomato cultivars and chemical control is not effective [5]. Bacteria can survive in soil and organic debris for several years, so preventive practices are the best alternative for its control, because bacterial dispersion is favored by cultural practices and irrigation [6,7]. The generation of fast, sensitive and cost-efficient methods is necessary for early disease detection. The European and Mediterranean Plant Protection Organization (OEPP/EPPO) has proposed a standard diagnostic protocol to isolate and identify the Cmm bacterium from plants and seeds [3]. The advantages and disadvantages considering the sensitivity and specificity of each diagnostic method, are discussed in [8,9,10]. The standard methods to diagnose and identify the causal agent Cmm involve bacterial isolation from the host tissue and its growth in a semi-selective media. Then bacterial colonies with suspicious cultural morphology are purified and analyzed by biochemical, serological, or molecular methods [3]. The diagnostic procedure is usually labor intensive, costly, and requires specialized technical training and scientific equipment.

BCTo disease detection in the field is currently based on the visual search of typical symptoms in the plant, such as the unilateral wilting of leaflets and leaves, the presence of corky spots on the stems and petioles, marginal leaf necrosis and internal stem discoloration, which can lead to sudden plant wilting and death [9]. However, the number of infected plants is usually higher than those with visible symptoms, and in many cases, the contagionʼs incidence is underestimated because Cmm can produce latent asymptomatic infections [7,11]. Previous works have found evidence that Cmm behaves as an endophyte during early stages of infection but, over time, the bacterium changes its behavior inducing disease symptoms. The transition from endophyte to pathogen is governed by the expression of putative virulence factors and the evasion or suppression of plant defense reactions. The phenomenon is modulated by bacterial population density (quorum-sensing), plant age, genetic diversity and the prevalent environmental conditions [4,8,9,10].

Raman spectroscopy (RS) is a spectroscopic technique used to determine the vibrational modes of molecules by recording the inelastic scattering of photons. A major technological innovation occurred with the invention of solid-state continuous wavelength lasers and highly stable CCDs that allowed Raman spectrometer miniaturization and portability. Currently, RS is implemented in multiple research areas, and its application in plant pathology are focused on early disease detection, because RS can differentiate diseased plants based on the biochemical changes induced by a specific phytopathogen during the plant-pathogen interaction [12]. RS procedures are fast, non-invasive and does not require previous sample preparations [13]. Several plant diseases with diverse etiology have been already studied using RS with the intention of performing an early plant disease diagnostic: *Candidatus Liberibacter asiaticus* and *Xanthomonas axonopodis* pv. *citri* bacterial pathogens in orange [*Citrus* x *sinensis* (L.) Osbeck] [14,15,16], pepper mild mottle virus (PMMoV) and Obuda pepper virus (ObPV) in chili crops (*Capsicum annuum* L.) [17]; Tomato yellow leaf curl Sardinia virus (TYLCSV) and tomato spotted wilt virus (TSWV) in tomato cultivars (*S. lycopersicum*) [18], the fungus *Aspergillus flavus*, *A. niger*, *Fusarium* spp., and, *Diplodia* spp., affecting maize grain (*Zea mays* L.) [19], the abutilon mosaic virus (AbMV) affecting abutilon (*Abutilum hybridum* Voss) cultivars [20] and the diagnosis of rose rosette disease (*Rosa* spp.) caused by the rose rosette virus (RRV) which is transmitted by eriophyid mites [21], among others.

Considering the aforementioned, the present research aim consists on the implementation of RS as a method for the early detection of the bacterial canker of tomato (BCTo), and thus, being able to differente the asymptomatic diseased plants from the healthy ones. At the same time, we also performed the quantitative validation of this technique as a preventive, rapid and accurate diagnostic tool.

## 2. Materials and Methods

### 2.1. Experimental Setup

Tomato seeds (*S. lycopersicum*) of Seminis^®^ Reserva F1 variety (Bayer AG, Leverkusen, Germany) were germinated in peat moss (Premier Horticulture Inc., Rivière-du Loup, QC, Canada), transplanted and kept in plastic containers. Subsequently, tomato plants at the four-leaf stage were infected with Cmm bacteria according to the procedure described by [5] to compose the infected plants treatment (BCTo). The healthy control plants (HTo) were inoculated with sterilized water under the same experimental conditions. Each treatment was on sets of ten plants and the experiment was repeated under the same conditions in order to verify the observed results. Cultural practices were done in accordance to local recommendations [22] and growth temperature was 23 ± 2 °C.

### 2.2. Raman Spectra Data Acquisition

Treatments were analyzed 30 days after the inoculation (DAI). The procedure consisted of randomly selecting three asymptomatic leaves from each asymptomatic plant. Leaf samples with no nutrient deficiency symptoms, chlorosis, physical damage, nor strange particles such as residues from insects or dust were selected. The leaves were rinsed with deionized sterile water and once dried leaflets were cut, and immediately analyzed at two-four points on the adaxial side. The Raman spectra were recorded by using a Horiba XploRA ONE™ confocal microscope spectrometer (Horiba Scientific, Ltd., Minami-ku, OP, Japan) equipped with a 785 nm DPSS laser, CCD photodetector and 2 cm^−1^ of spectral resolution. The Raman measurement conditions were 800–1800 cm^−1^ of spectral range, 10 s of acquisition time, 5 accumulations, ≈20 mW laser power, 1200 gr/mm grating, 100 μm slit, 300 μm hole and 20x magnification objective (micro spot with 10 μm ø). The calibration was performed daily by recording the Raman signal of a silicon wafer. In total, 177 spectra were obtained from infected plants (BCTo) and 120 spectra from the healthy control plants (HTo). Raman spectra shown in this work correspond to the raw baseline corrected results.

### 2.3. DNA Confirmatory Diagnosis

Bacterial isolation was done from vascular tissue of each tomato plant studied. The extracted tissue (0.5 g) was immersed in 20 mL of saline solution (NaCl 0.85%) and later 50 μL of the suspension were seeded in Petri dishes with yeast peptone glucose agar (YPGA) solid medium, and incubated at 28 °C. The isolated colonies with typical morphology to Cmm were identified by using 16S rRNA gene sequencing [3,23]. Single bacterial colonies were grown in NBY broth (nutrient broth 8 g/L, yeast extract 2 g/L, glucose 5 g/L, MgSO4 7H2O 0.24 g/L) for 24 h at 28 °C. The bacterial cell pellets were harvested (centrifuged at 12,000 rpm/10 min) and resuspended in lysis buffer (Tris-HCl 50 mM, EDTA 50 mM, SDS 3%), and incubated at 65 °C for 40 min. The lysates were mixed with an equal volume of phenol:chloroform:isoamyl alcohol (25:24:1) and centrifuged at 13,000 rpm for 5 min. The aqueous phases were then transferred to a new Eppendorf tube and mixed with 100 μL sodium acetate (3 M) and 1 mL of cold ethanol (−20 °C). The mixes were incubated on ice for 25 min and centrifuged at 13,000 rpm for 15 min. The supernatants were decanted, the DNA pellets were washed with 500 μL of ethanol 70% and centrifuged at 13,000 rpm for 2 min. The ethanol was decanted, and the DNA pellets were dried at room temperature. The pellets were resuspended in 50 μL of sterile Milli-Q^®^ water and stored at −20 °C. A fragment of 16S rRNA gene was PCR amplified using the universal primers F27 (5′-AGAGTTTGATCMTGGCTCAG-3′) and R1492 (5′-TACGGYTACCTTGTTACGACTT-3′) [24]. PCR products of expected sizes were purified and sequenced. The partial sequences of 16S rRNA genes were analyzed using Basic Local Alignment Search Tool Ver. 2.9.0 (BLASTn) at National Center for Biotechnology Information (NCBI, Bethesda, MD, USA) to identify strains for similarity comparison at the DNA sequence level [25].

### 2.4. Chemometric Analysis

In order to have an automated characterization of the spectral features from healthy (HTo) and asymptomatic infected plants (BCTo), the original spectra were first preprocessed by eliminating the background fluorescence by subtracting a fifth-order polynomial [26]. Once the baseline correction was done, a standard normal variate (SNV) normalization was applied taking in all the available data samples [27]. SNV normalization is given by applying the following equation:

(1) $$sn_{i}=\frac{s_{i}-\bar{s_{i}}}{\sqrt{\sum_{1}^{N}{\left(s_{i}-\bar{s_{i}}\right)}^{2}/\left(N-1\right)}}$$

where $sn_{i}$ is the entry corresponding to the *i-*th wavenumber (Raman shift) of the normalized vector, which correspond to the *i-*th Raman shift of each spectral raw data vector after the detrending process by subtraction of the fifth-order polynomial; it stands for the mean of all vectors and represents the total number of samples. Previous works [28,29] proposed to normalize Raman spectra to specific vibrational bands, because spectral intensities can vary with coloration of each specimen, and symptoms induced by biotic (phytopathogens) or abiotic (nutrient deficiencies) factors [30]. However, BCTo disease is characterized by the lack of symptoms during early disease stages, so we use SNV normalization due to the particularities of the analyzed pathosystem [10].

After data was prepossessed, we implemented the following sequence of algorithms using the Python programing language [31] for a mechanized feature extraction procedure. Starting by computing the first principal components (PCs) [32] of the set of vectors obtained by normalizing the debased numeric values of Raman spectra for the samples. These PCs were calculated by employing the Sklearn scientific programming libraries [33], as well as the corresponding fractions of explained variances for each PC. Once these data were obtained, with the intention of determining the spectral wavenumbers that have the greatest influence over the PCs, we developed a mechanism for the inspection of the coefficients of the correlation matrix obtained by the factor analysis procedure [34]. The criterion used to select the most important wavenumber for the classification was to consider those which’s associated correlation factors (loadings) satisfy the following inequality:

(2) $$\frac{2}{N}\overset{n}{\underset{i=1}{\sum }}\left|c_{i}\right|\sigma_{i}\geq \left|c_{max}\right|$$

where $c_{i}$ is the correlation between the PCi with the Raman shift to be analyzed, $\sigma_{i}$ is the explained variance for PCi, and $\left|c_{max}\right|$ represents the maximum of the absolute values of the correlation matrix. In this way, we manage to assign the largest weight to the correlations associated with the PCs that explain most of the variance among the spectral samples. With this set of wavenumber values, we created a sequence of continuous intervals, which were related to the vibrational modes of the compounds that differentiate the plant samples into the HTo (healthy) and BCTo (asymptomatic infected) classes. These spectrum areas give the main wavenumber intervals that reveal the largest contributions to the variances between both types of samples. The procedures described above convert such differences into a comparative metric for the quantitative description of the intervals with most influence in the classification. With the purpose of providing predictive capabilities to this methodology to differentiate their health condition (BCTo or HTo), we employed a classifier implemented by a neural network of the multilayer perceptron (MLP) type [35] employing the Sklearn library. A model with 1000 hidden layers was used with the logistic function acting as activation rule. To train the neural network, the Raman spectra were divided into two subsets. By doing so, 70% of the samples were used as a training set, the remaining 30% of the spectra were used for validation, such dataset division is commonly employed in artificial intelligence (AI) training stages in order to avoid overfitting [36] and as it is influenced by the proportion rather than the number of samples taken. Additionally, a classic classifier based on internal and external class variations was employed. The method of linear discriminant analysis (LDA) [37] was added in order to have comparative metrics and to ensure that the classification is not dependent on the classifier method, but on the information contained in the spectral samples. In the two classification models employed on this research, PCA is used for feature extraction, and then a classifier is applied over such features represented by the principal components after the criterion stated in Equation (2) is applied. Thus, comparing performances on disease detection with each of the two classifiers operating on the principal components of the spectra, we executed both procedures in combination with the principal component analysis (PCA). We identify each mechanism as (PCA + MLP) when a neural network is being used on the principal components and as (PCA + LDA) for the classifier using the principal components with the linear discriminant analysis. For each classifier we evaluate their performance with sensitivity values (SENS), specificity (SPEC), accuracy (ACC), positive prediction values (PPV), negative prediction values (NPV) and F1-Score. The equations employed in terms of true positives (TP), false positives (FP), true negatives (TN) and false negatives (FN) are described in [38].

To make sure that classifier agreement results are not influenced by chance, we evaluated the Cohen’s kappa coefficient [39] given by:

(3) $$K=\frac{\left(p_{o}-p_{e}\right)}{\left(1-p_{e}\right)}$$

where $p_{o}$ is the measured agreement between classifications and $p_{e}$ represents the probability for the classifications to be in agreement.

## 3. Results and Discussion

The group of infected plants (BCTo) remained asymptomatic during the evaluation, and they only displayed a general growth reduction as described by [40]. Bacterial colonies were isolated in YPGA medium from the BCTo group, and these colonies were circular, convex, with smooth edges and mucoid texture, opaque, of yellowish color and Gram (+) bacillus. Such features correspond to the ones described for Cmm [3]. The isolated strains were identified by sequencing a > 1 kilobase (kb) of genomic region 16S rRNA gene fragment and searching for homology at DNA level with BLASTn in NCBI. The analysis allowed to classify the isolated strains as related to *Clavibacter michiganensis* subsp. *michiganensis* when comparing with the reference sequences (NCBI Accession Number: HQ144239.1, KR922121.1, KR922121.1, HQ144230.1). No bacterial colonies similar to Cmm were isolated from control plants (HTo).

Bacterial isolation and DNA analysis (standard diagnostic protocol) confirmed the plants health condition: asymptomatic infected (BCTo) and healthy (HTo), so Raman spectroscopy is used to determine changes in plant metabolism of both groups, to provide a confirmatory diagnostic of the bacterial canker of tomato disease induced by Cmm. Previous studies have demonstrated that RS can allows to rapid confirmatory diagnostic, before symptoms onset [18,29].

### 3.1. Spectral Differences between Groups: HTo and BCTo

**Figure 1:**
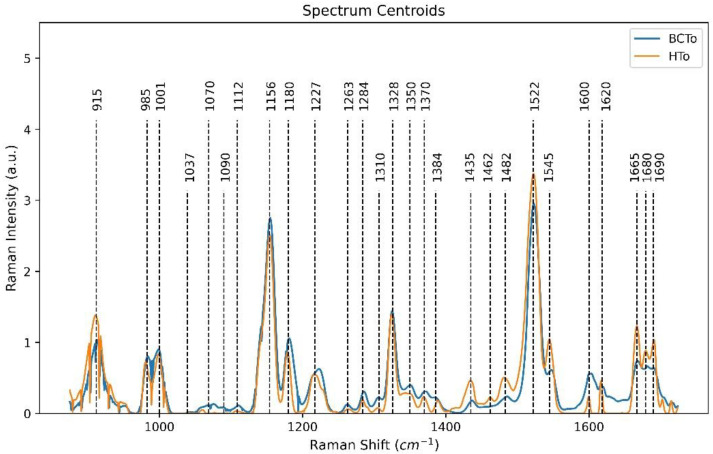
Normalized Raman spectra obtained from healthy tomato plants (HTo) and asymptomatic infected plants (BCTo). Dashed lines represent the wavenumber associated with key compounds.

The Raman spectra obtained from tomato leaves of healthy (HTo) and infected plants (BCTo) exhibited peaks associated to cellular components, and the most prominent vibrational bands were associated to monomeric and polymeric carbohydrates, carotenoids, chlorophyll, and phenolic compounds (Table 1). The compatible pathogen-plant interaction (Cmm-tomato) is an extremely complex biological system, because Cmm can manipulate the plant metabolism and evade defense responses allowing the bacteria to multiply and to shift from an endophytic to a pathogenic state, the process induce latent asymptomatic infections [10]. Biochemical alterations induced during the tomato-Cmm interaction were also detectable in the Raman spectral patterns (Figure 1).

BCTo group displayed an increased relative intensity at 1037–1090 cm^−1^ region, and bands of 1070 and 1310 cm^−1^, which are related to cellulose polymers [41,42]. The 1112 cm^−1^ wavenumber showed a higher relative intensity, the band is associated to carbohydrates monomer [18]; which suggests possible degradation processes on cell walls [4]. Different enzymes produced by Cmm take part in the degradation of cell walls during the infection process and these are responsible for the virulence of the pathogen [9,10].

**Table 1:** Vibrational bands and their assignments for tomato leaves.

Band (cm^−1^)	Vibrational Mode	Molecular Assignation
915	ν(C-O-H) in plane, symmetric	Cellulose, lignin [41]
985	δ(CH3)	Chlorophylls [43]
1001	δ(C-CH3)	Carotenoids [44,45]
1037–1090	CC and CO stretching	Cellulose [42]
1070	ν(CO)	Cellulose [41]
1112	δ(C-OH)	Carbohydrates [18]
1156	ν(C-C)	Carotenoids [44,45]
1180	ν(C-C) γ(CH)	Chlorophylls [18,46]
1227	δ(CCH)	Cuticle Triterpenoids [41]
1263	(=CH)	Carotenoids [18]
1284	δ(phenyl-OH)	Phenolics [44]
1310	δCH_2_ bending	Cellulose [41]
1328	δ(CH)·ν(CN)	Chlorophylls, Pyrrole ring br.- [18]
1350	CH_3_ Bend	Chlorophylls [46]
1370	C-H deformation (asymmetric)	Lignin [47]
1384	δCH_2_ bending	Aliphatic [48]
1435	δ(CH_2_), δ(CH_3_)	Cuticle Triterpenoids [48]
1462	δ(CH_2_), δ(CH_3_)	
1482	δ(CH_2_), δ(CH_3_)	
1522	ν1(C=C)	Carotenoids [44]
1545	C=O stretching	Flavonoids [49]
1600	C=C (aromatic ring)	Lignin [47]
1620	β sheet	Proteins [50]
1665	β sheet	Amide I [51,52]
1680	Cycl. [ν(C=O)]	Flavonoids [49]
1690	β turn	Proteins [50]

Diverse reports state that Cmm-infected plants accumulate phenolic compounds, such as lignin and callose, at their xylem tissues [53,54], the lignin are cross-linked phenolic polymers that also form part of cell walls, the associated bands exhibited an enhancement at 1370 and 1600 cm^−1^ [47]. Other Raman bands assigned to phenolic nature compounds also showed changes in the relative intensity, because the 1284 cm^−1^ wavenumber increased in diseased plants (BCTo). Flavonoids are substances considered with antimicrobial activity [53]. The healthy tomato group (HTo) showed higher intensity at 1545 and 1680 cm^−1^ wavenumbers, both Raman bands associated to flavonoids [49], which can be considered as indicators of Cmm infection during the asymptomatic stage.

Triterpenoids are compounds commonly found in plant cuticular wax, which have important functions, such as protection against pathogens [41,48]. The bands associated to triterpenoid compounds at 1227 [41], 1435, 1462 and 1482 cm^−1^ [48] showed an intensity difference between HTo and BCTo groups. Histological studies reported by [55] have stated that tomato plants infected by Cmm showed changes in the deposition of waxy substances on epidermal cells. Later, the thickening and lignification of cell walls were observed in infected plants. Changes on Raman bands associated with triterpenoids are important indicators of Cmm-infected plants during the asymptomatic stage.

Plants have a basal system for defense, and a second system based on signals that induce the activation of genes to deal with infections in progress [56,57]. Cmm has the ability of inhibiting these defense mechanisms in the tomato plants, because peroxidases and other enzymes are generally downregulated, while maintaining the functional photosynthetic activity [58,59]. In addition, during the oxidative stress induced by the infection process [58], the carotenoid compounds reduce the concentration of oxygen reactive species (ROS) [60] and, consequently, the stability of cell membranes and photosynthetic activity are maintained [61]. The representative chlorophyll Raman bands (985, 1180 and 1350 cm^−1^) remained more or less stable, only slightly increasing their relative intensity in the infected plants group (BCTo). The bands associated with carotenoid compounds showed similar behavior between the analyzed groups (HTo and BCTo), and these were represented by wavenumbers of 1001, 1156 and 1263, but the band at 1522 cm^−1^ has higher intensity in the healthy group (HTo).

Finally, changes were detected in the intensities of 1665 and 1690 cm^−1^ in the healthy tomato group (HTo), which can be assigned to proteins [50,51,52], this change probably indicates the plant long distance signaling transduction triggered by the biotic stressor (Cmm bacteria), characterized by the down-regulation of genes at early stages of Cmm infection [59].

The previously described observations match the biochemical changes induced by Cmm in tomato plant [8,9,10]. Previous works have demonstrated that RS techniques provide a clear differentiation between biotic stress (bacterial infection), abiotic stress (nutritional deficiencies) and healthy plants [16]. RS has the sensitivity to differentiate a simple or multiple viral infection in the same host [18,62], including the presence of secondary infections [30]. Additional works are required to determine the Raman spectral specificities to the different diseases that affect tomato plant.

### 3.2. Chemometric Analysis and Spectral Classification

**Figure 2:**
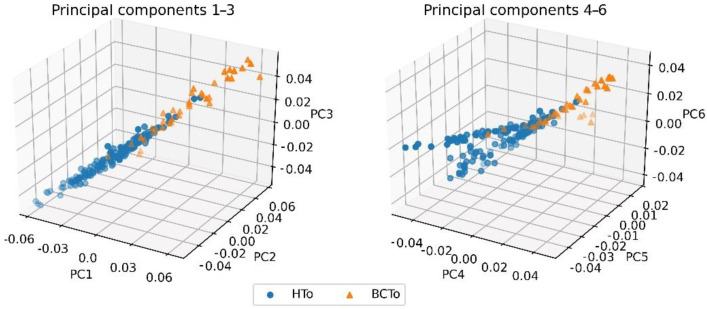
Class separability between HTo and BCTo sets across principal component spaces PC1–PC3 and PC4–PC6.

By using the PCA methodology, components of the six eigenvalues with the largest absolute contributing values to the spectral differences were obtained. These first 6 PCs explained up to 82% of the variance in the Raman spectra of the analyzed samples. The choice of use of PCA for feature extraction is based on the fact that the most spectral samples are linearly separable by hyperplanes in the component spaces [32] defined by PC1 to PC3 and PC4 to PC6 (Figure 2).

**Figure 3:**
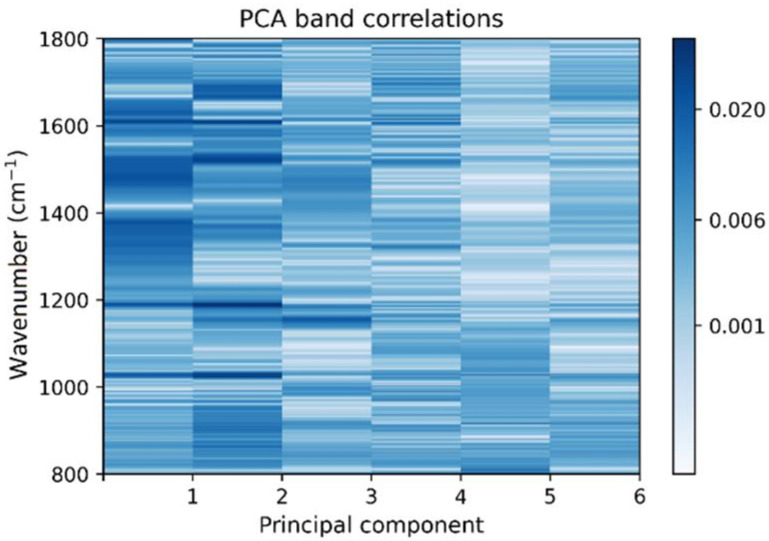
Correlation matrix for principal components (PCs) and the contribution (loading) of each wavenumber of the Raman spectra to the PC.

The absolute loading values are represented in Figure 3, they show which wavenumbers have bigger correlations with the features (in our case, principal components) that make the largest distinctions between Raman spectra corresponding to healthy (HTo) and the asymptomatic tomato infected plants (BCTo). The correlation matrix involved in the PCA process was examined under the procedure described in the methodology section of this document to automatically detect the spectral band intervals, which are associated to chemical species of interest (Table 1) considered as disease markers [18].

The main wavenumbers in the first principal components, can be thought as the ones implicated with the changes of Raman intensity bands associated to the biochemical compounds that distinguish the infected plants from healthy ones [18,30]. Therefore, the largest loadings and the associated wavenumbers with their respective related chemical compounds. Although, there are other features not presented with large influence in the correlation matrix for the first PCs (Figure 3), such characteristics cannot be directly associated to changes of Raman intensity bands associated to biochemical compound of interest. The larger correlation values (0.01–0.02) were associated to compounds from the photosynthetic system (chlorophyll and carotenoids), the cell walls components (cellulose and lignin) and other substances considered as antimicrobial metabolites (phenolic and flavonoids). It is worth noting that while Figure 1 depicts only the centroids of the spectra for the two HTo and BCTo sets, their respective variances are not considered in the figure. Therefore, it is difficult in this case, if not impossible, to effectively make a characterization of the two groups by only considering spectral centroids. Such centroids are not used by the feature extraction procedure employed here. Instead, a combination of PCs and linear discriminant loading-based criteria determined by Equation (2) is used. Variances are best analyzed along principal component axes, and that is the justification for which PCA are taken as input for the proposed classifier. On the other hand, we found that, considering principal components, the spectra samples exhibited great class separability between the two groups on the feature spaces regarding PC1–3 and PC 4–5, depicted at Figure 2. It is important to note that, in comparison with the centroids of the spectra shown in Figure 1, where some important bands present similar average values, as should be expected due to the fact that most infected plants were asymptomatic, PC vectors plotted in Figure 2 are distributed in a more separable manner. This separability comes from the different behavior presented by the variances of each group along the considered range of the spectra for both classes and is quantitatively computed by the LDA method. To make sure that such separability correlates to BCTo-associated compounds, the automated technique described in the methodology section was executed, the results showed that many of the wavenumbers that have larger contributions to the main PCs, corresponded to chemical compound associated to the compatible pathogen-plant interaction as described in the previous section.

The classifiers algorithms multilayer perceptron (PCA + MLP) and linear discriminant analysis (PCA + LDA) can differentiate the classes analyzed (HTo and BCTo). Performance of the PCA + MLP and PCA + LDA classifiers presented high sensitivity values in both cases (1.0), but the specificity was superior for PCA + MLP (0.95) compared to the PCA + LDA (0.88) classifier. The accuracy and positive predictive value were slightly higher in PCA + MLP (0.99 and 0.98) when compared to PCA + LDA (0.97 and 0.97), respectively. The metric parameters NPV and F1-score showed adequate performance for both classifiers (1.0 and 0.99). Performance measurements for the classification strategies employed in this works are shown in Table 2. The few cases that cannot be easily distinguished in a linear fashion (LDA), the multilayer perceptron (MLP) can operate over features in a non-linear way, which is probably the reason for its superior performance. Therefore, the steps described in the sections above provide a way to detect asymptomatic infected plants, by means of Raman spectroscopy.

Cohen’s kappa coefficients were obtained between the two classifiers PCA + MLP and PCA + LDA ($\kappa_{mlp-lda}$) and for each of them with accordance to the ground truth $\left(\kappa_{mlp-gnd}\right)$ and $\left(\kappa_{lda-gnd}\right)$ respectively according to Equation (3). Table 3 shows the coefficients resulted from each calculation.

All values shown in Table 3 are close to 1, which means that the agreement on class labels obtained is not influenced by chance, and that the result is not dependent on the classifier. Besides, having $\left(\kappa_{mlp-gnd}\right)\;$as the value nearest to 1, confirms that the method using the neural network is more accurate with respect to the ground truth than the more traditional PCA + LDA approach.

## 4. Conclusions

Early detection of bacterial canker of tomato is essential for epidemiological monitoring and plant disease management. The present work has demonstrated the usefulness of Raman spectroscopy as an efficient, fast and reliable technology to differentiate the infected asymptomatic plants (BCTo) from their healthy counterparts (HTo), by means of Raman spectral signatures obtained from their developing leaves. The biochemical changes induced by the plant-pathogen interaction were detectable by Raman spectra and separable by machine-learning. The multilayer perceptron (MLP) and linear discriminant analysis (LDA) algorithms showed reliable performance values after data treatment by means of principal component analysis (PCA), but PCA + MLP was slightly compared to PCA + LDA. High accuracy in discerning asymptomatic infected plants from healthy ones with the procedures here described comes from the fact that the principal components of the spectra belonging to each group are distributed across highly separable sets on the principal component space. Despite having, at first glance, very similar spectral centroids, their respective variances differ significantly among spectral frequency bands, identified in this work by the wavenumbers that have larger contributions to the main PCs. These bands are associated the vibrational modes of key chemical compounds associated to the disease process, and such conditions were exploited for an effective detection of the infected plants with Cmm bacteria.

## Figures and Tables

**Table 2:** Classifier performance comparison for characterization of Raman spectra of healthy (HTo) and asymptomatic infected plants (BCTo).

Metrics	PCA + MLP	PCA + LDA
SENS	1.0	1.0
SPEC	0.95	0.88
ACC	0.99	0.97
PPV	0.98	0.97
NPV	1.0	1.0
F1-Score	0.99	0.99

SENS = Sensitivity, SPEC = Specificity, ACC = Accuracy, PPV = Positive Predictive Value, NPV = Negative Predictive Value.

**Table 3:** Cohen’s kappa coefficients (*κ*) comparing agreement.

*κ_mlp−lda_*	*κ_mlp−gnd_*	*κ_lda−gnd_*
0.937326	0.922452	0.985053

Subscripts: mlp = multilayer perceptron; lda = linear discriminant analysis; gnd = ground truth

## Data Availability

The data that support the findings of this study are available from the corresponding author upon reasonable request.
